# Supplementary material for: An improved nuclei isolation protocol from leaf tissue for single-cell transcriptomics
Source: PLoS One. 2025 Sep 10;20(9):e0302118. doi: 10.1371/journal.pone.0302118 (PMC12422464; doi:10.1371/journal.pone.0302118)
Supplement: S1 Table — (PDF) [file pone.0302118.s001.pdf]

S1 Table - Cell-type-specific markers for cell type identification of maize leaf snRNA-seq.

Gene markers for cell GeneID	Cell type	Gene name
Zm00001eb038930	mesophyll	*MDH6*
Zm00001eb158810	mesophyll	*CAH1*
Zm00001eb383680	mesophyll	*PEPC1*
Zm00001eb121470	bundle sheath	*ME3*
Zm00001eb092540	bundle sheath	*SSU2*
Zm00001eb197410	bundle sheath	*SSU1*
Zm00001eb073420	guard cell	*SLAC1*
Zm00001eb344960	guard cell	*FAMA*
Zm00001eb288410	subsidiary cell	*SWEET1b*
Zm00001eb158420	subsidiary cell	*KCH1*
Zm00001eb336530	epidermis	*VT2*
Zm00001eb070050	epidermis	*HB75*
Zm00001eb233310	Vasculature	*GLK53*
Zm00001eb397900	Vasculature	*LIMTF13*
