# Supplementary material for: An improved nuclei isolation protocol from leaf tissue for single-cell transcriptomics
Source: PLoS One. 2025 Sep 10;20(9):e0302118. doi: 10.1371/journal.pone.0302118 (PMC12422464; doi:10.1371/journal.pone.0302118)
Supplement: S2 Table — (PDF) [file pone.0302118.s002.pdf]

S2 Table – Primer sequences

**Primer name**	**Sequence**
atpA F2	TCAGAATCAATTGGCAAGG
atpA R2	CCTCTCGTTCCGGTATAAA
psbA F2	ATACCCAGACGGAAACTAA
psbA R2	CGGTCCTTATGAGCTAATTG
EIF1a-F	TGGGCCTACTGGTCTTACTACTGA
EIF1a-R	ACATACCCACGCTTCAGATCCT
UBQ7-F	CAGACTACAACATCCAGAAG
UBQ7-R	TATTAGACGACGACATCCATA
