# Supplementary material for: An improved nuclei isolation protocol from leaf tissue for single-cell transcriptomics
Source: PLoS One. 2025 Sep 10;20(9):e0302118. doi: 10.1371/journal.pone.0302118 (PMC12422464; doi:10.1371/journal.pone.0302118)
Supplement: S3 Table — (PDF) [file pone.0302118.s003.pdf]

S3 Table. Number of cells per cluster for each sample

Cluster	With filter	Without filter
0	392	508
1	440	372
2	361	382
3	53	636
4	227	168
5	91	104
6	76	80
7	26	73
8	33	36
9	10	21
**Total**	**1709**	**2380**
