# Supplementary figures and images for: An improved nuclei isolation protocol from leaf tissue for single-cell transcriptomics
Source: PLoS One. 2025 Sep 10;20(9):e0302118. doi: 10.1371/journal.pone.0302118 (PMC12422464; doi:10.1371/journal.pone.0302118)

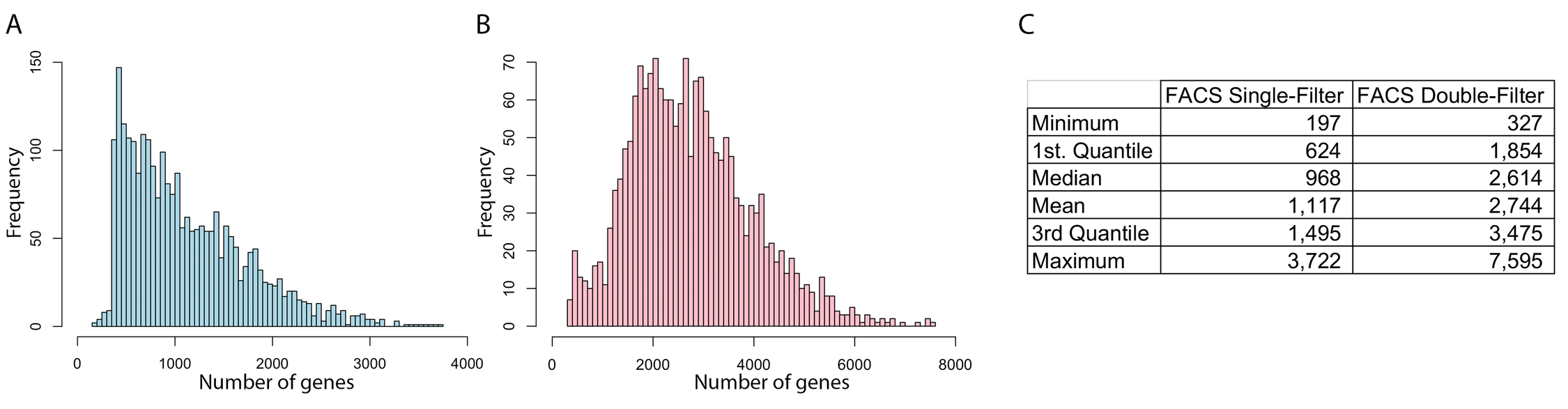

Supplement: S1 Fig — (A) Histogram of the number of genes per nucleus in the sample prepared with the FACS single-filter strategy (higher chloroplast contamination). (B) Histogram of the number of genes per nucleus in the sample prepared with the FACS double-filter strategy (lower chloroplast contamination). (C) Summary of the quantile summary of the number of genes per nucleus. (TIFF) [file pone.0302118.s004.tif]

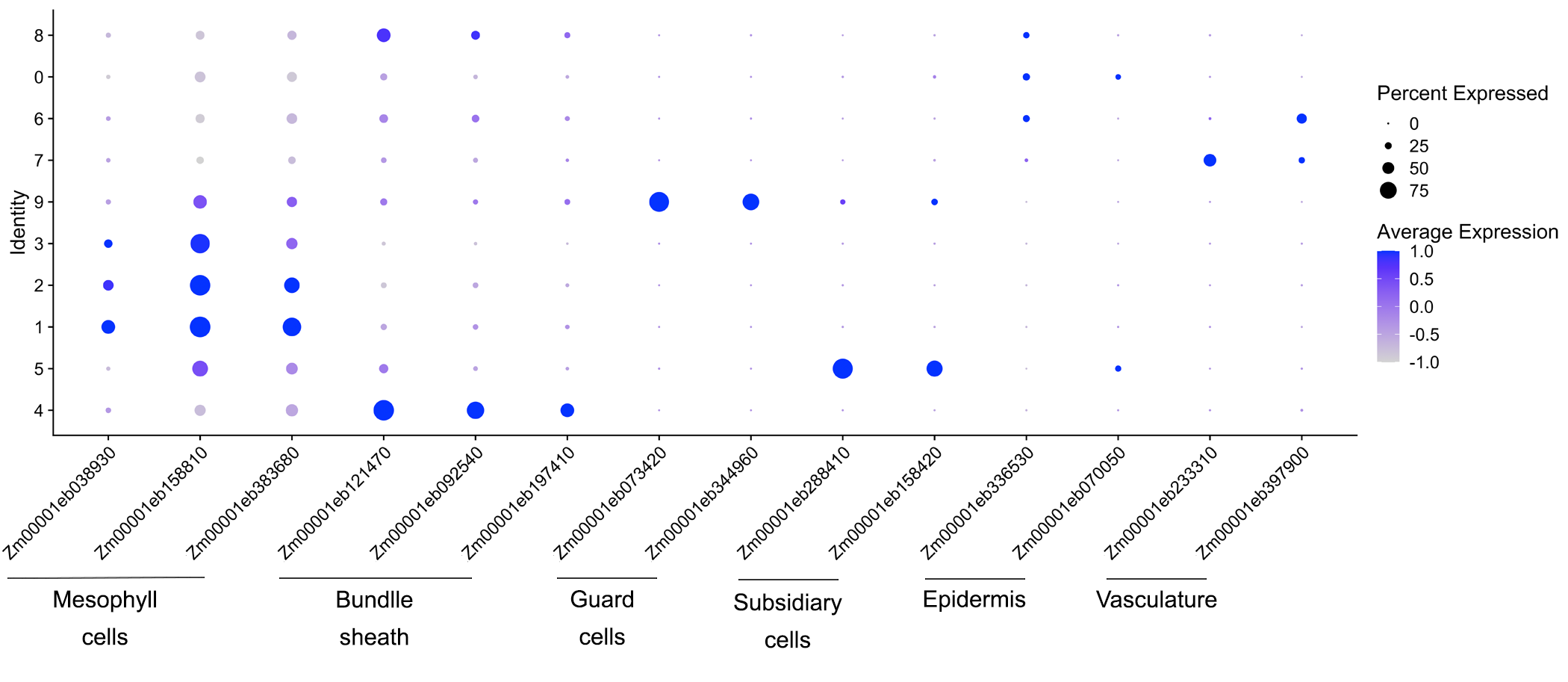

Supplement: S2 Fig — (TIFF) [file pone.0302118.s005.tif]

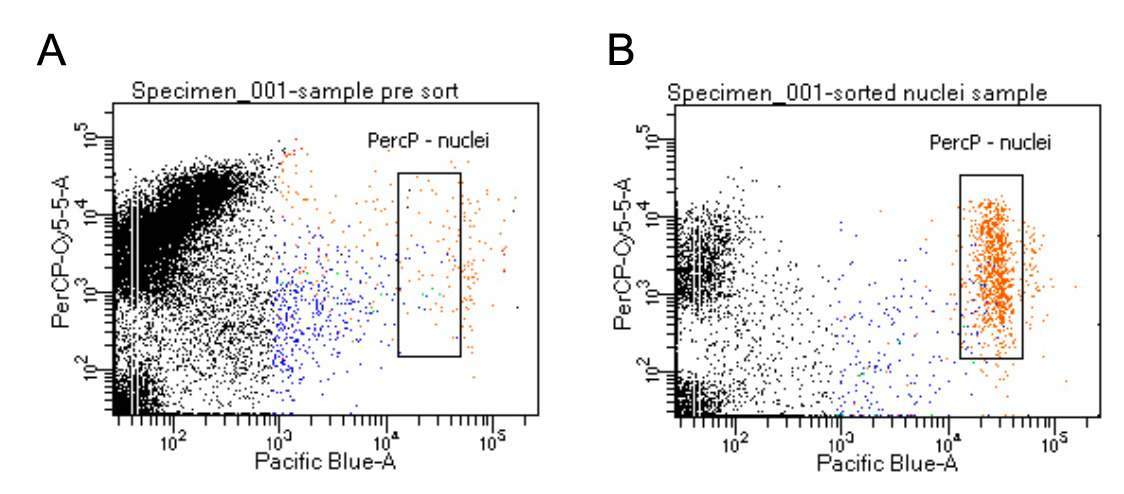

Supplement: S3 Fig — (A) Dot-plot of PerCP and DAPI positive events. The black dots indicate PerCP-positive particles. (B) Dot-plot of PerCP and DAPI positive events. Orange dots correspond to DAPI-positive events. (TIFF) [file pone.0302118.s006.tif]
