# Supplementary material for: An improved nuclei isolation protocol from leaf tissue for single-cell transcriptomics
Source: PLoS One. 2025 Sep 10;20(9):e0302118. doi: 10.1371/journal.pone.0302118 (PMC12422464; doi:10.1371/journal.pone.0302118)
Supplement: Attachment — Submitted filename: Combined_review_final.pdf [file pone.0302118.s007.pdf]

Overall comment:

The article illustrated a potential improvement on the traditional method of performing single-nucleus sequencing on maize leaves. It is a nice and straightforward method, and the application area is relevant, providing methodology development for a less investigated area of single cell/nuclei RNA sequencing (scRNA-seq or snRNA-seq). The findings and detailed methods could be very useful for many plant molecular biologists. Based on the logic behind filtering out chloroplasts based on their specific emission spectrum, I expect that this method should indeed lead to some improvements in the results, but unfortunately the extent of the improvement is not quantitated, and it is unclear from the current way the data is presented. I cannot recommend publication of the submission in its current form for several key reasons:

1) The present manuscript claims improvements in performance on the snRNA-seq data quality, but this claim is lacking strong support from data in the manuscript. Many statements use terms such as 'substantial' and 'significant' without support from actual statistics that is derived from the data. It will be important to show the improvement accurately, clearly, and with scientific rigor for publication.
2) The claims also appear to be largely based on a comparison with data in an article that has an incomplete citation ("Madrid et al., 2024", mentioned on line 111 without any further information) that is not available for the reviewer to assess. I cannot tell if the results figures in this manuscript use the same data from that Madrid paper or not, therefore without providing details on the sample itself, enough experiment replicates, and sufficient statistical analysis, it is hard to reach a consensus that this new reported method is better than the previous method.
3) The overall data reporting is lacking, and more analysis or comparisons using different metrics and methods will help to illustrate the advantages of this new method.
4) Some issues with linguistic accuracy that could mislead readers. For example, the word "cell" appears multiple times after the nucleus isolation step, which might be confusing. The word choice and grammar in the sentences need to be reviewed by the author, and conciseness can also be improved.

To elaborate:

Data/Results and Figures are not sufficiently informative/quantitative.

Only 4 figures of data, and two tables does not seem sufficient to justify or support the claim that the present 'improved method' is much better than the old one. I urge the authors to think more deeply about how to perform statistics on their datasets to

quantitatively highlight the improvement in the results. For example, for basic QC metrics, violin plots or elbow plots of the nuclei captured, and number of genes, UMI, or other features detected will be informative. For the FACS sorting results, were there any replicates? If no, then how can we assess the robustness of the method? If there are replicates, summarize statistics of events captured for both the ‘old’ and ‘new’ methods should be presented. Anecdotal/representative evidence such as traces from FACS plots etc should not be the only evidence used. The image quality is also very poor for the FACS plots. Microscopy images in Fig 2 lack labels on the images, e.g. what is being imaged, what are we supposed to be looking at? What are sub-figs A to G respectively? The scale bars are so tiny even in the full res downloaded version of the Figure, it is impossble to see what scale it is. While such information may be included in the figure caption or methods writeup, the authors should take some effort to make the figures easily understandable by the readers. Similar comments could be made for Fig 3 – are there replicates, and what are we looking at? Are they histograms? The legend is equally cryptic “with filter” “without filter” – what kind of filter? FACS filter? QC filter? Mapping filter? And these data should be more quantitative, such as the mode or median or mean UMI counts etc. There are so many sc and sn RNA-seq papers out there, many of them present different ways to show and assess quality control; the authors could choose some of those existing methods for quantitating data quality and report relevant statistics. Figure 4 suffers from the same lack of information. No indication of what samples are presented here; whether there are replicates, and the numbers of cells being shown in each (yes, I know it’s in the table, but the total n should be labeled in the UMAP as well for easy referencing). Especially since the authors want to point out that one method is ‘better’, they should illustrate this by indicating the proportions of cells captured in each case in the figure itself. The sample that each UMAP refers to should be clearly labeled in the figure, not only appear in the caption. Marker genes, or whatever was used to label/annotate the different cell types should also be clearly indicated. It’s also unclear why there are circles around some of the clusters, but not others – maybe it is to group similar cell types, but if this is the case, the authors should use color coding or proper legend labels. The clusters seem to not even use the same color scheme for the two datasets; cluster 6 is blue in one UMAP but green in another, and refer to different cell types in each. One UMAP has 13 clusters, while the other has 12. This is a confounding factor, because if a different resolution is being used for the two datasets, then how can the authors know that the difference in cell proportions or representation is due to the filtering method, and not the clustering or annotation method?

All in all, I strongly recommend that the authors can further study other sn/scRNA-seq papers on how to perform statistics and presentation of the data for effective illustration of the performance. While it is costly to perform replicates for the snRNA-seq part of the experiment, at least replicates should be done using FACS to generate

statistics demonstrating robustness. Alternatively, perhaps the authors could seek a collaborator with experience in these areas to consult/help with data analysis and presentation.

Misleading language

Starting from the title, the language is misleading, as single-nuclei transcriptomics is NOT the same as single-cell transcriptomics. The discussion of nuclei isolation in performing a single-**cell** transcriptomics study is inaccurate. This kind of mixing of terminology appears many times in the manuscript. For example, on Line 61: *"In plants, an approach aiming to cause less damage to cells during tissue dissociation is the use of nuclei over protoplasts for the cell isolation step."* The plant cell is the entirety of the structure enclosed by the cell wall, including the nucleus and the cytoplasm and other organelles. To retrieve the nuclei, it seems you would need to break open the cell wall and lose the rest of the cell. This sentence seems to suggest that isolation of the nuclei does not require cell lysis? Or that somehow the tissue dissociation for single nuclei would result in intact cells? It seems totally contradictory. This kind of misleading and confusing writing is found throughout the manuscript. The authors should carefully read through and make sure the terminology used is accurate throughout – making clear distinction between the use of the whole cell, the isolated nuclei, and references to other organelle, such as the removal of chloroplasts. If this description is due to some unique anatomy of the maize leaf, then a diagram should be included for the reader's understanding.

Grammar and punctuation should also be further improved throughout (e.g. there are missing punctuation and conjunctions throughout the manuscript).

Further detailed comments:

Line 22: *Arabidopsis thaliana* is not a close relative of maize; it seems awkward to specifically point to this species here if this manuscript is focusing on maize. Perhaps it would be better to discuss root vs leaf tissue processing in general, or use the comparison of "commonly used model species *A. thaliana*" vs "less commonly used species" as the parallel instead. It would make the writing more logical. Furthermore, the authors should use, or at least mention, the proper taxonomic name of the species they are using, rather than just the common name maize. The genome reference *Z. mays* is mentioned only in the methods section on mapping, but throughout the rest of the manuscript it is never mentioned.

Line 28: "we achieved... a reduction in the number of cells removed due to quality control issues" – this could be misleading, bioinformaticians will set their own

thresholds for QC and removal of low quality cells. It may be better to instead say that “we achieved... a reduction in the number of low quality cells”, assuming that the authors did indeed use the same QC metrics when comparing the before/after filtering datasets. While we are on this topic, it is important to indicate clearly what were the metrics/criteria used to remove ‘low quality cells’, and clearly mention whether the same metrics were used to compare both protocols (old vs new).

Lines 29-30: Was it not possible to identify every cell type in the maize leaf prior to the invention of this filtering method? From Fig 4 it seems this is not the case; old protocol also identifies the same cell types, just with different proportion. This sentence could also be misleading.

Lines 29-32: This protocol kept being referred to as the “improved” or “enhanced” protocol but there was never a mention about the “original” protocol. Some re-writing of the text could remedy this and make the discussion more clear.

Line 36-39: There should be citation of sources for these other scRNA-seq studies.

Line 44: language: "responses of plants to environmental cues"

Lines 45-47: The first two sentences sound a little bit contradictory. So is it that “scRNA-seq success” depends on the dataset’s quality, or that the dataset’s quality is dependent on the “scRNA-seq success”? I suggest collapsing them into one clear and concise sentence.

Line 49: Sample preparation includes cell isolation. Use Tissue processing instead of sample preparation would be more appropriate.

Lines 49-51: Citation is needed.

Lines 51-52: I think it would be better to elaborate the previous statement than to introduce a new statement that sounds similar.

Lines 45-55: Maybe in this paragraph, the authors can give a concrete example of how a poor or misleading “dataset” was obtained due to mistakes made during tissue handling, and/or the other factors mentioned here? It is also not clear how this exactly relates to reduction of statistical power – while experts in the field do know, it would be better to explicitly state the relationship, i.e. too many cells of low quality will be unusable for analysis and therefore the total cell number available for doing statistics will be low.

Line 68: “Afterwards,” and “whole cells”

Line 68: “Afterwards”

Line 70: What is meant by “common” step?

Lines 71-76: I suggest putting these sentences together with the next paragraph since they are on the same topic, namely, the issues and challenges of nuclei isolation.

Lines 85-88: This sentence sounds quite long-winded, redundant, and a bit unclear (especially the "counted along with the cell" part). I suggest rephrasing the sentence and breaking it up into two as such: "These cell-free RNAs may be mistakenly included as part of an intact cell's RNA, confounding the identity of the original cell. This may lead to the erroneous merging of two distinct cell populations during downstream analyses."

Line 111: I suggest rephrasing this sentence to "lower than the expected 70% genome alignment rate for RNA-seq reads". But also, the Conesa et al. paper mentioned the 70% as what is expected for humans. And the Schmitz paper did not really mention a specific genome alignment percentage that is expected for plant RNA reads. The only mention of expected genome alignment in that paper is that it must be consistent and not low. But no specific number mentioned. It may be better to rephrase this part so as not to misrepresent the cited work.

Line 112: "This *number* is a key indicator of library quality, a low alignment value indicates possible problems with RNA quality." Does low alignment value also affect the usability of the data? If so, perhaps can include that in addition to just the "RNA quality"? Also, the authors might need to cite a source that says genome alignment rate is a key indicator of library quality.

Lines 113-115: How was the determination done? Is there a source that can be cited? Was it in the previous study? If so, the authors will need to cite that study here.

Line 116: So what is really novel about this method? Earlier in line 71 it was mentioned that FACS is part of a "common" step? I assume "common" there meant it was commonly included as part of the protocol? Being more precise will help to highlight the improvements.

Line 122: I suggest rephrasing to "Compared to our previous results, results generated using this method boasts improved library quality and a reduction in the number of discarded low-quality cells." Additionally, similar to line 28, it may be more precise to stick with mentioning just how many low quality cells are affected, so perhaps the word "discarded" can be discarded (pun intended).

Line 124: This sentence can connect with the previous one better by adding "Lastly, our approach also did not generate bias..."

Lines 125-126: This sentence has too many "for"s. I suggest rephrasing to "These results suggest that our protocol has the potential to enhance single-nuclei RNA sequencing experiments in leaves."

Line 130: Three plants as in three leaves? How many leaf sections used in total, and they came from how many leaves? It might be good to specify.

Lines 144-150: Maybe start with the positive selection of DAPI-stained nuclei before the negative selection just to make the order of the information clearer since that was the order it was mentioned in in the line 143 sentence.

Line 161: total reads obtained (gross number) should be presented, not just the target for mapping. Especially since in the results section, only percentages were shown.

Line 166: I suggest rephrasing it to "...and concatenated both to the B73v5 genome." for clarity's sake.

Line 193: Similar to line 112, it might be necessary to specify that the presence of these plastids can also affect data usability / introduce biases. Saying that it affects the QC process makes it sound less important because the QC criteria are subject to change.

Lines 193-194: It sounds a little bit inaccurate to say that pure and intact nuclei was extracted "in silico" because nuclei extraction is a physical process. Perhaps it is more accurate to say "Data collected during FACS allows for the extraction of pure and intact single nuclei and smooth *in silico* processing of snRNA-seq data."

Line 197: Similar to line 146, I suggest adding "wavelengths" after "emit".

Lines 124-125: There is no statistics or any other quantitative assessment of bias (what does ground truth look like?); as such I do not think the authors can claim that there is "no bias".

Line 128: Vital information readers might look for is missing. The origin, the breed, and the growth environment of the maize plants should be included. How are the plants chosen, was it through random selection? Is there a specific reason behind choosing the V5 leaves stage over other stages? Any replicates for this experiment? The midsection here stands for the region between the base and the tip, since there is no clear boundary among these three regions, is there a way to illustrate how you pick this midsection? How did you store those "sections"?

Lines 135-136: This sentence isn't grammatically correct. If the sentence is simplified, we would get that "the nuclei were separated and repeated" which doesn't make sense.

Line 146: It's not necessarily the cell population that's removed right?

Lines 171-174: Is normalizing this way appropriate? It would be great if you could share some details regarding the previous scRNA-seq dataset. What is the reason behind a lower number of reads?

Lines 178-179: Is it transcripts per cell? Since only the nucleus has been sequenced.

Line 186: Do you mean the nucleus sequence that contains over 0.5% of the chloroplast gene sequence was excluded?

Line 186: Does that mean a percentage higher than 0.5% is considered as having a "larger amount of chloroplast contamination"? I think it's better to add a citation to explain the reasons behind this. Also, are there other sequences removed? For example, cells with few detected genes should be removed as well. At the same time, It has been reported that chloroplast genes can transfer to the nucleus (Huang et al., 2003). Is the 0.5% here a consensus?

Line 201: How was it determined? Please include more detail.

Line 205: "*were* gated"

Lines 213-214: It is overclaiming to say the sample is free of cellular debris and plastid contamination, since the FACS plot shows an overlap in the spectra of the two populations. It is also unlikely that ALL cellular debris have been removed. Such absolute claims should be avoided; using language such as 'after filtering, we did not observe cellular debris via visual inspection" or "XXX analysis indicate a reduction by % of plastid DNA contamination" are more suitable.

Line 233: Quantitative assessment of the reduction is needed, it's not enough to just say "greatly reduced" based on qualitative judgement. Perhaps cell counting can be performed along with simple statistics like ANOVA.

Lines 235-241: What about plot E? It is missing from the figure description.

Lines 245-246: What does "especially for reads mapped to the genome" mean? I thought the "read alignment" mentioned here refers to genome mapping? Might need to rephrase this sentence to make it clearer.

Lines 249-250: So how many reads are achieved in total? It's better to add this information. Please be specific what metrics are increased (e.g. use the same wording used previously; sequencing metrics). Does the sample in the previous study have a comparable amount of chloroplast? Probably need to quantify the density of chloroplast in the samples from both studies.

Line 252: It seems like the number is still below the 70% goal. Any statistical evidence or articles support that this is a substantial change? How do we prove that the differences are not due to batch effect?

Line 255 (Table 1): It is not immediately obvious what "filter" is since the authors never referred to the FACS method as a "filter". Perhaps the labels can be changed to "with FACS" and "without FACS".

Lines 265-267: If there are low quality cells identified here as a separate peak, should they be removed too as part of the quality control process?

Line 272: Similar to line 252, how is this reduction considered “significant” and what effect does this have on downstream analyses?

Lines 272-273: This could also be due to the batch effect. More replicates are needed.

Line 285: How “similar” is it really? Again, please include quantitative metrics.

Lines 286-287: “All the expected cell types” – please provide references and some discussion of these known cell types in the introduction or results section. In my experience, most fields continue to uncover new cell types in familiar tissue types, as the number of sc/snRNA-seq replicates and number of cells being analyzed increases. I would suggest not using such absolute/cavalier language because there could certainly be cell types in maize leaf that have not been identified yet due to technical or other reasons.

Lines 302-307: I think this part has already been covered in the introduction.

Line 312: Is there a way to check the data loss and sequencing quality or library complexity in this study in addition to the metrics that were already assessed? It seems like those are quite important metrics, so assessing those metrics and presenting them will really highlight the improvements that this method can bring.

Lines 312-314: It's better to explain more about this part and show the citation

Line 323: How does one define “very low percentage”? If a threshold is chosen, rationale for choosing the threshold should be discussed and justified. It should not be arbitrary.

Line 346-347: I do not see evidence of downstream analysis being done, therefore the claim that there is no impact is unfounded.

Lines 352-353: The reference cited here actually mentioned that there’s more chloroplasts in bundle sheath cells in maize.

Line 363: I do not have access to this ‘previous work’ and cannot assess these claims. Observed variations and batch effects can be addressed by replicates and using statistics; scientifically we cannot just claim that variations are not due to bias in the method but due to biological differences. To establish a new method, this is exactly what is needed from the authors: to measure the bias of the method in the context of naturally occurring biological variation. If the bias or variation in the method itself cannot be quantified, then how can one do analysis to compare different biological samples?
