# Supplementary material for: An improved nuclei isolation protocol from leaf tissue for single-cell transcriptomics
Source: PLoS One. 2025 Sep 10;20(9):e0302118. doi: 10.1371/journal.pone.0302118 (PMC12422464; doi:10.1371/journal.pone.0302118)
Supplement: Attachment — Submitted filename: Responses to reviewers.docx [file pone.0302118.s009.docx]

**Responses to reviewers**

Thank you very much for all the comments and suggestions. We are addressing individual concerns below. The answers to all the points are in blue.

**Overall comment:**

The article illustrated a potential improvement on the traditional method of performing

single-nucleus sequencing on maize leaves. It is a nice and straightforward method,

and the application area is relevant, providing methodology development for a less

investigated area of single cell/nuclei RNA sequencing (scRNA-seq or snRNA-seq). The

findings and detailed methods could be very useful for many plant molecular biologists.

Based on the logic behind filtering out chloroplasts based on their specific emission

spectrum, I expect that this method should indeed lead to some improvements in the

results, but unfortunately, the extent of the improvement is not quantitated, and it is

unclear from the current way the data is presented. I cannot recommend the publication of

the submission in its current form for several key reasons:

1) The present manuscript claims improvements in performance on the snRNA-seq

data quality, but this claim is lacking strong support from data in the manuscript.

Many statements use terms such as ‘substantial’ and ‘significant’ without

support from actual statistics that is derived from the data. It will be important to

show the improvement accurately, clearly, and with scientific rigor for

publication.

We agree with the reviewer, and we have added quantitative data and analysis to support the conclusion of the paper. More details about these analyses and data are described and explained below.

2) The claims also appear to be largely based on a comparison with data in an

article that has an incomplete citation (“Madrid et al., 2024”, mentioned on line

111 without any further information) that is not available for the reviewer to

assess. I cannot tell if the results figures in this manuscript use the same data

from that Madrid paper or not, therefore without providing details on the sample

itself, enough experiment replicates, and sufficient statistical analysis, it is hard

to reach a consensus that this new reported method is better than the previous

method.

Because the paper in which the data from the snRNA-seq library using the old method (single filter – without removing the chloroplast in the FACS step) is still under review, we decided to make this data available in this manuscript.

3) The overall data reporting is lacking, and more analysis or comparisons using

different metrics and methods will help to illustrate the advantages of this new

method.

Thanks for the comment. We have added more analysis using different metrics, such as the number of UMIs, the number of genes, and the number of cells above the chosen quality threshold. More details about these analyses and data are described and explained below.

4) Some issues with linguistic accuracy that could mislead readers. For example,

the word “cell” appears multiple times after the nucleus isolation step, which

might be confusing. The word choice and grammar in the sentences need to be

reviewed by the author, and conciseness can also be improved.

Thanks for the suggestion. We agree that the use of the word cell instead of nuclei may mislead the readers. We have made the corrections in the revised manuscript.

To elaborate:

**Data/Results and Figures are not sufficiently informative/quantitative.**

Only 4 figures of data and two tables do not seem sufficient to justify or support the

claim that the present ‘improved method’ is much better than the old one. I urge the

authors to think more deeply about how to perform statistics on their datasets to quantitatively highlight the improvement in the results. For example, for basic QC

metrics, violin plots or elbow plots of the nuclei captured, and number of genes, UMI, or

other features detected will be informative.

Thanks for the suggestion. We have added more tables and figures to present the effectiveness of our improved method and to compare it with the old one. As mentioned above, we have included and compared more QC metrics between the two methods, such as the number of UMIs, the number of genes, and the number of cells above the chosen quality threshold. Violin plots (Fig. 4) have been added to show the comparison of standard quality control metrics (Number of transcripts per cell, Number of genes identified per cell, Percentage of chloroplast-encoded genes per cell) between the two methods.

For the FACS sorting results, were there any replicates? If no, then how can we assess the robustness of the method? If there are replicates, summarize statistics of events captured for both the ‘old’ and ‘new’ methods should be presented. Anecdotal/representative evidence such as traces from FACS plots etc should not be the only evidence used. The image quality is also very poor for the FACS plots.

In the original manuscript, we did not present replicate data for the FACS results. Data for replicates have been added to the revised manuscript. Based on these data, we now present more evidence of the effectiveness of our improved method, as can be seen in Figure 6. By repeating the FACS step three times, we were able to summarize statistics of events captured for both methods (Fig.6A – counting of the number of chloroplasts and nuclei in samples generated with double filter strategy [new method] and single filter strategy [old method]). We also performed an RT-qPCR in the samples acquired using both methods to check for the expression of chloroplast-encoded genes (Fig.6B).

Microscopy images in Fig 2 lack labels on the images, e.g. what is being imaged, what are we supposed to be looking at? What are sub-figs A to G respectively? The scale bars are so tiny even in the full res downloaded version of the

Figure, it is impossible to see what scale it is. While such information may be included in

the figure caption or methods writeup, the authors should take some effort to make the

figures easily understandable by the readers.

Thanks for the suggestion. We agree with the reviewer and have changed this figure (Fig. 3 in the revised manuscript) to make it more readable.

Similar comments could be made for Fig 3

– are there replicates, and what are we looking at? Are they histograms? The legend is

equally cryptic “with filter” “without filter” – what kind of filter? FACS filter? QC filter?

Mapping filter? And these data should be more quantitative, such as the mode or

median or mean UMI counts etc. There are so many sc and sn RNA-seq papers out

there, many of them present different ways to show and assess quality control; the

authors could choose some of those existing methods for quantitating data quality and

report relevant statistics.

We have changed this Figure (Fig.4 in the revised to manuscript) to violin plots to better show the QC metrics.

Figure 4 suffers from the same lack of information. No

indication of what samples are presented here; whether there are replicates, and the

numbers of cells being shown in each (yes, I know it’s in the table, but the total n should

be labeled in the UMAP as well for easy referencing). Especially since the authors want

to point out that one method is ‘better’, they should illustrate this by indicating the

proportions of cells captured in each case in the figure itself. The sample that each

UMAP refers to should be clearly labeled in the figure, not only appear in the caption.

Thanks for the suggestion; we added labels to Fig.4 (Fig.5 in the revised paper). The proportion of number of cells per cluster (for each method) is presented in Table 2. The total number of cells per cluster (for each method) is presented in S3 Table.

Marker genes, or whatever was used to label/annotate the different cell types should

also be clearly indicated.

Markers used to annotate the clusters are shown in S1 Table and S1 Fig.

It’s also unclear why there are circles around some of the clusters, but not others – maybe it is to group similar cell types, but if this is the case, the authors should use color coding or proper legend labels.

Thanks for the suggestion. The circles were removed from the samples, and we are using color coding to properly legend the labels.

The clusters seem to not even use the same color scheme for the two datasets; cluster 6 is blue in one UMAP but green in another, and refer to different cell types in each. One UMAP has 13 clusters, while the other has 12. This is a confounding factor, because if a different resolution is being used for the two datasets, then how can the authors know that the difference in cell proportions or representation is due to the filtering method, and not the clustering or annotation method?

We agree with the reviewer and have made appropriate changes. We have now applied the same resolution in the analysis of both data sets. We have also integrated both data sets to remove any technical bias (See material and methods for more details about data integration).

All in all, I strongly recommend that the authors can further study other sn/scRNA-seq

papers on how to perform statistics and presentation of the data for effective

illustration of the performance. While it is costly to perform replicates for the snRNAseq

part of the experiment, at least replicates should be done using FACS to generate

statistics demonstrating robustness. Alternatively, perhaps the authors could seek a

collaborator with experience in these areas to consult/help with data analysis and

presentation.

As mentioned above, we have included replicates to show the robustness of our improved method using the FACS double filter strategy.

**Misleading language**

Starting from the title, the language is misleading, as single-nuclei transcriptomics is

NOT the same as single-cell transcriptomics. The discussion of nuclei isolation in

performing a single-cell transcriptomics study is inaccurate. This kind of mixing of

terminology appears many times in the manuscript. For example, on Line 61: “In

plants, an approach aiming to cause less damage to cells during tissue dissociation

is the use of nuclei over protoplasts for the cell isolation step.” The plant cell is the

entirety of the structure enclosed by the cell wall, including the nucleus and the

cytoplasm and other organelles. To retrieve the nuclei, it seems you would need to

break open the cell wall and lose the rest of the cell. This sentence seems to suggest

that isolation of the nuclei does not require cell lysis? Or that somehow the tissue

dissociation for single nuclei would result in intact cells? It seems totally

contradictory. This kind of misleading and confusing writing is found throughout the

manuscript. The authors should carefully read through and make sure the

terminology used is accurate throughout – making clear distinction between the use

of the whole cell, the isolated nuclei, and references to other organelle, such as the

removal of chloroplasts. If this description is due to some unique anatomy of the

maize leaf, then a diagram should be included for the reader’s understanding.

Grammar and punctuation should also be further improved throughout (e.g. there are

missing punctuation and conjunctions throughout the manuscript).

Thanks for the recommendations. Changes have been made through the text to address these points.

**Further detailed comments:**

**Line 22:** Arabidopsis thaliana is not a close relative of maize; it seems awkward to

specifically point to this species here if this manuscript is focusing on maize.

Perhaps it would be better to discuss root vs leaf tissue processing in general, or use

the comparison of “commonly used model species A. thaliana” vs “less commonly

used species” as the parallel instead. It would make the writing more logical.

Furthermore, the authors should use, or at least mention, the proper taxonomic

name of the species they are using, rather than just the common name maize. The

genome reference Z. mays is mentioned only in the methods section on mapping, but

throughout the rest of the manuscript it is never mentioned.

We agree with the reviewer and have removed the sentence about *A. thaliana*. We now focused more on the challenges in working with leaf samples (tissue with high chloroplast content – See lines 23-31). In addition, we added the proper taxonomic name of maize earlier in the abstract (See line 31).

**Line 28:** “we achieved… a reduction in the number of cells removed due to quality

control issues” – this could be misleading, bioinformaticians will set their own thresholds for QC and removal of low quality cells. It may be better to instead say that

“we achieved… a reduction in the number of low quality cells”, assuming that the

authors did indeed use the same QC metrics when comparing the before/after filtering

datasets. While we are on this topic, it is important to indicate clearly what were the

metrics/criteria used to remove ‘low quality cells’, and clearly mention whether the

same metrics were used to compare both protocols (old vs new).

Thanks for the observation. We agree with the reviewer and have removed this sentence. Both data sets were analyzed with the same QC metrics. All the QC metrics used in the analysis were added in the material and methods.

**Lines 29-30:** Was it not possible to identify every cell type in the maize leaf prior to the

invention of this filtering method? From Fig 4 it seems this is not the case; old protocol

also identifies the same cell types, just with different proportion. This sentence could

also be misleading.

The sentence was removed in the revised manuscript.

**Lines 29-32:** This protocol kept being referred to as the “improved” or “enhanced”

protocol but there was never a mention about the “original” protocol. Some re-writing of

the text could remedy this and make the discussion more clear.

It is an improved version of the most used currently available protocol for nuclei extraction for single-nuclei RNA-seq experiments (References 9 and 13).

9. Thibivilliers S, Anderson D, Libault M. Isolation of Plant Root Nuclei for Single Cell RNA Sequencing. Curr Protoc Plant Biol. 2020;5. doi:10.1002/cppb.20120

13.	Conde D, Triozzi PM, Balmant KM, Doty AL, Miranda M, Boullosa A, et al. A robust method of nuclei isolation for single-cell RNA sequencing of solid tissues from the plant genus Populus. PLoS One. 2021;16: e0251149.

**Line 36-39:** There should be citation of sources for these other scRNA-seq studies.

Citations were added (References 1 to 5).

**Line 44:** language: "responses of plants to environmental cues"

The sentence was rewritten. (See lines 45-46).

**Lines 45-47:** The first two sentences sound a little bit contradictory. So is it that “scRNAseq

success” depends on the dataset’s quality, or that the dataset’s quality is

dependent on the “scRNA-seq success”? I suggest collapsing them into one clear and

concise sentence.

The sentence was rewritten. (See lines 47-49).

**Line 49:** Sample preparation includes cell isolation. Use Tissue processing instead of

sample preparation would be more appropriate.

Thanks for the suggestion. Changes were made, see line 50.

**Lines 51-52:** I think it would be better to elaborate the previous statement than to

introduce a new statement that sounds similar.

We kindly disagree with the reviewer and think the sentences should stay the way they were written.

**Lines 45-55:** Maybe in this paragraph, the authors can give a concrete example of how a

poor or misleading “dataset” was obtained due to mistakes made during tissue

handling, and/or the other factors mentioned here? It is also not clear how this exactly

relates to reduction of statistical power – while experts in the field do know, it would be

better to explicitly state the relationship, i.e. too many cells of low quality will be

unusable for analysis and therefore the total cell number available for doing statistics

will be low.

Thank you. We have added some language to expand on the reduction of statistical power. While we are not aware of a specific dataset where this comparison was performed, we do include citations from the Single-cell Best Practices Consortium, acknowledging that low-quality cells can be a problem.

**Line 68:** “Afterwards,” and “whole cells”

Changes were made. See line 70.

**Line 70:** What is meant by “common” step?

The word common was removed from the sentence. See lines 71-72.

**Lines 71-76:** I suggest putting these sentences together with the next paragraph since

they are on the same topic, namely, the issues and challenges of nuclei isolation.

We kindly disagree with the reviewer and think the sentences should stay in the current paragraph.

**Lines 85-88:** This sentence sounds quite long-winded, redundant, and a bit unclear

(especially the “counted along with the cell” part). I suggest rephrasing the sentence

and breaking it up into two as such: “These cell-free RNAs may be mistakenly included

as part of an intact cell’s RNA, confounding the identity of the original cell. This may

lead to the erroneous merging of two distinct cell populations during downstream

analyses.”

Thanks for the suggestion. Changes were made. See lines 86-88.

**Line 111:** I suggest rephrasing this sentence to “lower than the expected 70% genome

alignment rate for RNA-seq reads”. But also, the Conesa et al. paper mentioned the 70%

as what is expected for humans. And the Schmitz paper did not really mention a

specific genome alignment percentage that is expected for plant RNA reads. The only

mention of expected genome alignment in that paper is that it must be consistent and

not low. But no specific number mentioned. It may be better to rephrase this part so as

not to misrepresent the cited work.

We removed this part of the manuscript to avoid misrepresentation of the cited work.

**Line 112:** “This number is a key indicator of library quality, a low alignment value

indicates possible problems with RNA quality.” Does low alignment value also affect the

usability of the data? If so, perhaps can include that in addition to just the “RNA

quality”? Also, the authors might need to cite a source that says genome alignment rate

is a key indicator of library quality.

This part has been removed from the introduction.

**Lines 113-115:** How was the determination done? Is there a source that can be cited?

Was it in the previous study? If so, the authors will need to cite that study here.

We aligned the un-mapped reads to the maize chloroplast genome. The sentence was changed in the manuscript to add this information (see lines 291-292).

**Line 116:** So what is really novel about this method? Earlier in line 71 it was mentioned

that FACS is part of a “common” step? I assume “common” there meant it was

commonly included as part of the protocol? Being more precise will help to highlight the

improvements.

Changes were made through the text to make clear the innovations of our method, which includes the FACS double filter strategy.

**Line 122:** I suggest rephrasing to “Compared to our previous results, results generated

using this method boasts improved library quality and a reduction in the number of

discarded low-quality cells.” Additionally, similar to line 28, it may be more precise to

stick with mentioning just how many low quality cells are aRected, so perhaps the word

“discarded” can be discarded (pun intended).

Sentences were rewritten for clarity (See lines 113-119).

**Line 124:** This sentence can connect with the previous one better by adding “Lastly, our

approach also did not generate bias...”

This part was removed from the manuscript.

**Lines 125-126:** This sentence has too many “for”s. I suggest rephrasing to “These

results suggest that our protocol has the potential to enhance single-nuclei RNA

sequencing experiments in leaves.”

Thanks for the suggestion. Changes were made. See lines 118-119.

**Line 130:** Three plants as in three leaves? How many leaf sections used in total, and

they came from how many leaves? It might be good to specify.

One leaf section from three plants was pooled. The information was added in the revised manuscript. See 124-125.

**Lines 144-150:** Maybe start with the positive selection of DAPI-stained nuclei before the

negative selection just to make the order of the information clearer since that was the

order it was mentioned in in the line 143 sentence.

Thanks for the suggestion. We changed the previous sentence to: We used a double filter strategy to negatively select autofluorescent chloroplast and positively select DAPI-stained nuclei.

**Line 161:** total reads obtained (gross number) should be presented, not just the target

for mapping. Especially since in the results section, only percentages were shown.

**Line 166:** I suggest rephrasing it to “…and concatenated both to the B73v5 genome.” for

clarity’s sake.

Thanks for the suggestion. Changes were made in the revised manuscript. See lines 178-179.

**Line 193:** Similar to line 112, it might be necessary to specify that the presence of these

plastids can also affect data usability / introduce biases. Saying that it affects the QC

process makes it sound less important because the QC criteria are subject to change.

Thanks for the suggestion. Changes were made in the revised manuscript. See lines 238-239.

**Lines 193-194:** It sounds a little bit inaccurate to say that pure and intact nuclei was

extracted “in silico” because nuclei extraction is a physical process. Perhaps it is more

accurate to say “Data collected during FACS allows for the extraction of pure and intact

single nuclei and smooth in silico processing of snRNA-seq data.”

Thanks for the suggestion. Changes were made in the revised manuscript. See lines 238-239.

**Line 197:** Similar to line 146, I suggest adding “wavelengths” after “emit”.

Thanks for the suggestion. Changes were made in the revised manuscript. See line 242.

**Lines 124-125:** There is no statistics or any other quantitative assessment of bias

(what does ground truth look like?); as such I do not think the authors can claim that

there is "no bias".

We believe there is no bias because the same cell types with similar proportions of cells per cell type were found in the data generated with both methods.

**Line 128:** Vital information readers might look for is missing. The origin, the breed,

and the growth environment of the maize plants should be included. How are the

plants chosen, was it through random selection? Is there a specific reason behind

choosing the V5 leaves stage over other stages? Any replicates for this experiment?

The midsection here stands for the region between the base and the tip, since there

is no clear boundary among these three regions, is there a way to illustrate how you

pick this midsection? How did you store those "sections"?

Thank you. We have added these descriptions. We collected leaves from V5 maize because they correspond to a mature source leaf, and we reasoned that this would be a good stage to explore the sugar metabolism in maize leaf, which our lab is interested. Yes, plants were chosen randomly from the ones that were at the selected growth stage on collection day. The midsection was selected by visual determination of the middle of the leaf between base and tip.

**Lines 135-136:** This sentence isn't grammatically correct. If the sentence is

simplified, we would get that "the nuclei were separated and repeated" which

doesn't make sense.

The sentence was rewritten. See line 141.

**Line 146:** It's not necessarily the cell population that's removed right?

Thanks for the observation. The words cell population were replaced by events. See line 150.

**Lines 171-174:** Is normalizing this way appropriate? It would be great if you could

share some details regarding the previous scRNA-seq dataset. What is the reason

behind a lower number of reads?

The total number of reads is a consequence of the amount of sequencing resources spent and the efficiency of the specific sequencing run, which leads to differences from library to library. To make sure that the comparison was not biased by sequencing amounts, which is a parameter that the user can control. We believe that it is appropriate for the specific method comparison.

**Lines 178-179:** Is it transcripts per cell? Since only the nucleus has been sequenced.

Thanks for the observation. The word cell(s) was replaced by nucleus/nuclei. See line 198.

Line 186: Do you mean the nucleus sequence that contains over 0.5% of the

chloroplast gene sequence was excluded?

Yes, that is correct.

**Line 186:** Does that mean a percentage higher than 0.5% is considered as having a

"larger amount of chloroplast contamination"? I think it's better to add a citation to

explain the reasons behind this. Also, are there other sequences removed? For

example, cells with few detected genes should be removed as well. At the same

time, It has been reported that chloroplast genes can transfer to the nucleus (Huang

et al., 2003). Is the 0.5% here a consensus?

This number was chosen based on our observation of the number of unmapped reads aligning to the maize chloroplast genome. Unfortunately, we cannot cite any study because this step hasn’t been performed in any sc/snRNA-seq study. The chloroplast-encoded genes are removed bioinformatically from the data set. All the QC metrics, including cells with few genes were included in the material and methods.

**Line 201:** How was it determined? Please include more detail.

Based on the chlorophyll fluorescence.

Line 205: “were gated”

Thanks for the observation. Change was made. See line 249.

**Lines 213-214:** It is overclaiming to say the sample is free of cellular debris and

plastid contamination, since the FACS plot shows an overlap in the spectra of the

two populations. It is also unlikely that ALL cellular debris have been removed. Such

absolute claims should be avoided; using language such as ‘after filtering, we did not

observe cellular debris via visual inspection” or “XXX analysis indicate a reduction

by % of plastid DNA contamination” are more suitable.

We re-wrote the sentence. See lines 258-259.

**Line 233:** Quantitative assessment of the reduction is needed, it’s not enough to just

say “greatly reduced” based on qualitative judgement. Perhaps cell counting can be

performed along with simple statistics like ANOVA.

We have added quantitative assessment. See Figure 6.

**Lines 235-241:** What about plot E? It is missing from the figure description.

Thanks for the observation. Changes were made to address this. See line 273.

**Lines 245-246:** What does “especially for reads mapped to the genome” mean? I

thought the “read alignment” mentioned here refers to genome mapping? Might need to

rephrase this sentence to make it clearer.

The CellRanger software provides the percentage of alignment to the genome and transcriptome.

**Lines 249-250**: So how many reads are achieved in total? It's better to add this

information. Please be specific what metrics are increased (e.g. use the same wording

used previously; sequencing metrics). Does the sample in the previous study have a

comparable amount of chloroplast? Probably need to quantify the density of

chloroplast in the samples from both studies.

The total number of reads generated for both libraries was added to the revised manuscript (See lines 171-173).

Because the leaves used to extract the nuclei were from the same developmental stage and grown in the same conditions, and we used the same protocol to extract the nuclei (up to the FACS step), there is no reason why they shouldn’t have a comparable number of chloroplasts. In addition, we have added chloroplast and nuclei counting from three different replicates to the revised manuscript. While we didn’t specifically quantify the density of chloroplast, we believe the counting sufficiently addresses this point.

**Line 252:** It seems like the number is still below the 70% goal. Any statistical evidence

or articles support that this is a substantial change? How do we prove that the

differences are not due to batch effect?

We believe the chloroplast counting from three different independent replicates provides strong evidence that this difference is not due to batch effect. We agree that more replicates of snRNA-seq would provide additional evidence, but we believe this should be done in future studies considering the cost involved and the fact that the different sources of evidence provided here support the proposed method.

**Line 255 (Table 1):** It is not immediately obvious what “filter” is since the authors never

referred to the FACS method as a “filter”. Perhaps the labels can be changed to “with

FACS” and “without FACS”.

We changed to FACS single filter strategy and FACS double filter strategy.

**Lines 265-267:** If there are low quality cells identified here as a separate peak, should

they be removed too as part of the quality control process?

We changed the way to look at the QC metrics. But if one wants to use this previous way to look at the metrics, yes. If a separate peak is identified, one should remove the cells.

**Line 272:** Similar to line 252, how is this reduction considered “significant” and what

the effect does this have on downstream analyses?

Not only was the number of cells that passed the threshold higher in the data set generated using our method, but all the QC metrics were also higher (see lines 306-320).

**Lines 272-273:** This could also be due to the batch effect. More replicates are

needed.

Although we agree that the batch effect may have an effect on the results, we do believe that our observations are due to the effectiveness of our method. Single-cell experiments are extremely expensive (~$2,500 per sample). Our data from the replicates (number of chloroplast and nuclei as well as gene expression of chloroplast-encoded genes) are more evidence for the effectiveness of our method.

**Line 285:** How “similar” is it really? Again, please include quantitative metrics.

The number of clusters (cell types) and the proportion of cells per cluster show that both methods generated similar results regarding the identification of cell types.

**Lines 286-287:** “All the expected cell types” – please provide references and some

discussion of these known cell types in the introduction or results section. In my

experience, most fields continue to uncover new cell types in familiar tissue types,

as the number of sc/snRNA-seq replicates and number of cells being analyzed

increases. I would suggest not using such absolute/cavalier language because there

could certainly be cell types in maize leaf that have not been identified yet due to

technical or other reasons.

We agree with the reviewer and have changed the word expected by known.

**Lines 302-307:** I think this part has already been covered in the introduction.

Thanks for the observation, but we prefer to keep it in the discussion section as well.

**Line 312:** Is there a way to check the data loss and sequencing quality or library

complexity in this study in addition to the metrics that were already assessed? It seems

like those are quite important metrics, so assessing those metrics and presenting them

will really highlight the improvements that this method can bring.

We have included the quality scores of the sequencing run to represent sequencing quality metrics.

**Lines 312-314:** It's better to explain more about this part and show the citation.

More details were added in lines 431-434 in the revised manuscript.

**Line 323:** How does one define “very low percentage”? If a threshold is chosen,

rationale for choosing the threshold should be discussed and justified. It should not be

arbitrary.

As it is done for mitochondrial genes (to remove cells going through cell death), the choice of the threshold is a little bit arbitrary. As mentioned earlier, because this is the first time someone has paid attention to the chloroplast contamination (and not only bioinformatically removing the chloroplast-encoded genes), we don’t have any parameters that are mostly used (like the mitochondrial genes), and this is the threshold we used to eliminate most GEMs that contained chloroplast or chloroplast transcripts.

**Line 346-347:** I do not see evidence of downstream analysis being done, therefore the

claim that there is no impact is unfounded.

We rephrased this sentence.

Lines 352-353: The reference cited here actually mentioned that there’s more

chloroplasts in bundle sheath cells in maize.

Thanks for the observation. However, the study states the following: “ Using estimates for the length of cells from other sources (Table [S3](https://nph.onlinelibrary.wiley.com/doi/10.1111/nph.18956#support-information-section); Fig. [S7](https://nph.onlinelibrary.wiley.com/doi/10.1111/nph.18956#support-information-section)) and the count of chloroplasts per cell observed in our SBS-SEM images, we estimate maize has 53 chloroplasts in both cell types, and sugarcane has 49 chloroplasts per mesophyll cell and 62 chloroplasts per bundle sheath cell.”

What the authors observed was that in maize, chloroplasts occupied a higher cell volume in bundle sheath cells (60-70%) compared to the mesophyll cells (30-50%).

We have corrected this information in the revised manuscript (See lines 428-429).

**Line 363:** I do not have access to this ‘previous work’ and cannot assess these claims.

Observed variations and batch effects can be addressed by replicates and using

statistics; scientifically we cannot just claim that variations are not due to bias in the

method but due to biological differences. To establish a new method, this is exactly

what is needed from the authors: to measure the bias of the method in the context of

naturally occurring biological variation. If the bias or variation in the method itself

cannot be quantified, then how can one do analysis to compare different biological

samples?

This was already addressed in previous points.
